# ICAM5 as a Novel Target for Treating Cognitive Impairment in Fragile X Syndrome

**DOI:** 10.1523/JNEUROSCI.2626-18.2019

**Published:** 2020-02-05

**Authors:** Ya-ping Pei, Yue-yi Wang, Dan Liu, Hui-yang Lei, Zhi-hao Yang, Zi-wei Zhang, Man Han, Ke Cheng, Yu-shan Chen, Jin-quan Li, Gui-rong Cheng, Lang Xu, Qing-ming Wu, Shawn M. McClintock, Ying Yang, Yong Zhang, Yan Zeng

**Affiliations:** ^1^Brain Science and Advanced Technology Institute,; ^2^Hubei Province Key Laboratory of Occupational Hazard Identification and Control,; ^3^Big Data Science and Engineering Research Institute, Wuhan University of Science and Technology, Wuhan 430065, People's Republic of China,; ^4^Department of Psychiatry, UT Southwestern Medical Center, Dallas, Texas 75390,; ^5^Department of Pathophysiology, School of Basic Medicine and The Collaborative Innovation Center for Brain Science, Key Laboratory of Hubei Province and Ministry of Education of China for Neurological Disorders, Tongji Medical College, Huazhong University of Science and Technology, Wuhan 430030, People's Republic of China, and; ^6^Department of Neurobiology, School of Basic Medical Sciences and Neuroscience Research Institute, and Key Laboratory for Neuroscience, Ministry of Education of China and National Committee of Health and Family Planning of China, Peking University, Beijing 100191, People's Republic of China

**Keywords:** cognitive impairment, dendritic spine maturation, FMRP/ICAM5 mRNA interaction, fragile X mental retardation protein, fragile X syndrome, intercellular adhesion molecule 5

## Abstract

Fragile X syndrome (FXS) is the most common inherited form of intellectual disability, resulted from the silencing of the *Fmr1* gene and the subsequent loss of fragile X mental retardation protein (FMRP). Spine dysgenesis and cognitive impairment have been extensively characterized in FXS; however, the underlying mechanism remains poorly understood. As an important regulator of spine maturation, intercellular adhesion molecule 5 (ICAM5) mRNA may be one of the targets of FMRP and involved in cognitive impairment in FXS. Here we show that in *Fmr1* KO male mice, ICAM5 was excessively expressed during the late developmental stage, and its expression was negatively correlated with the expression of FMRP and positively related with the morphological abnormalities of dendritic spines. While *in vitro* reduction of ICAM5 normalized dendritic spine abnormalities in *Fmr1* KO neurons, and *in vivo* knockdown of ICAM5 in the dentate gyrus rescued the impaired spatial and fear memory and anxiety-like behaviors in *Fmr1* KO mice, through both granule cell and mossy cell with a relative rate of 1.32 ± 0.15. Furthermore, biochemical analyses showed direct binding of FMRP with ICAM5 mRNA, to the coding sequence of ICAM5 mRNA. Together, our study suggests that ICAM5 is one of the targets of FMRP and is implicated in the molecular pathogenesis of FXS. ICAM5 could be a therapeutic target for treating cognitive impairment in FXS.

**SIGNIFICANCE STATEMENT** Fragile X syndrome (FXS) is characterized by dendritic spine dysgenesis and cognitive dysfunctions, while one of the FMRP latent targets, ICAM5, is well established for contributing both spine maturation and learning performance. In this study, we examined the potential link between ICAM5 mRNA and FMRP in FXS, and further investigated the molecular details and pathological consequences of ICAM5 overexpression. Our results indicate a critical role of ICAM5 in spine maturation and cognitive impairment in FXS and suggest that ICAM5 is a potential molecular target for the development of medication against FXS.

## Abstract (precis)

Fragile X syndrome (FXS) is the most common inherited form of intellectual disability, resulted from the silencing of the *Fmr1* gene and the subsequent loss of fragile X mental retardation protein (FMRP). Spine dysgenesis and cognitive impairment have been extensively characterized in FXS; however, the underlying mechanism remains poorly understood.

## Introduction

Fragile X syndrome (FXS) is the most common inherited cause of mental retardation, resulted from the transcriptional silencing of the fragile X mental retardation 1 (*Fmr1*) gene and the subsequent loss of fragile X mental retardation protein (FMRP; Davis and Broadie, 2017). FXS is characterized by cognitive impairment associated with a broad spectrum of psychiatric comorbidities, including hyperactive behavior, autism spectrum disorder, poor attention, and seizure (Wang et al., 2012; Specchia et al., 2017). Currently, there is no effective therapy for treating cognitive impairment in FXS, making the quest for novel targets of considerable importance. A common neuropathologic phenotype seen in FXS is dendritic spine malformation, which is associated with an increased number of long, thin, and tortuous spines, and a pruning failure during spine transition from development to mature ones (Irwin et al., 2001; Portera Cailliau and Yuste, 2001; Wijetunge et al., 2014). Dysgenesis of dendritic spines is also observed in many other neurodevelopmental disorders (NDDs; Irwin et al., 2001; Portera Cailliau and Yuste, 2001; Calabrese et al., 2006; Bourgeron, 2009; Penzes et al., 2011; De Rubeis et al., 2012; Wijetunge et al., 2014). Thus, determining the molecular mechanisms involved in impaired dendritic spine formation and maturation and cognitive impairment may shed light on FXS and other NDDs for the development of new therapeutic strategies.

Under normal conditions, FMRP exists at a high level in dendritic spines (Antar et al., 2005; Davis and Broadie, 2017). Through binding to mRNAs, FMRP regulates the expression of many genes at the post-transcriptional level (Darnell and Klann, 2013; Specchia et al., 2017), including mRNA dendritic localization, axonal/dendritic transport, and export from the nucleus to the cytoplasm (Bassell and Warren, 2008; Melko and Bardoni, 2010; Pasciuto and Bagni, 2014). In the FXS pathological condition, FMRP loss-of-function results in excessive protein synthesis, and further defects in synaptic plasticity, as well as cognitive impairment (Bassell and Warren, 2008; Darnell and Klann, 2013). Over the last 20 years, a large number of potential mRNA targets of FMRP have been found (Brown and Huynh, 2001; Darnell et al., 2011; Ascano et al., 2012). A well known study proposed an underestimated list of 842 mRNA targets of FMRP and also listed a total of 19,493 related complexes, including intercellular adhesion molecule 5 (ICAM5; Darnell et al., 2011). However, the pathological consequences of these mRNA targets remain unclear.

ICAM5 is a cell adhesion molecule that belongs to the Ig superfamily (Yoshihara and Mori, 1994). It is widely expressed in telencephalic neurons and plays an important role in higher-order cognitive and motor functions (Mori et al., 1987; Oka et al., 1990; Mitsui et al., 2007). In the early postnatal life of mammals, the ontogenic appearance of ICAM5 is consistent with the timing of dendritic outgrowth, spine formation, and synaptogenesis, and its expression persists into adulthood (Mori et al., 1987; Yoshihara et al., 1994; Tian et al., 2000). In addition, the cleavage of ICAM5 leads to a reduced density of filopodia via β_1_ integrin interaction and the consequent phosphorylation of cofilin (Conant et al., 2011), decreases glutamatergic transmission and neuronal excitability (Niedringhaus et al., 2012), and alters spine maturation and learning performance (Nakamura et al., 2001). These findings suggest that ICAM5 is involved in dendritic spine morphogenesis and synapse development. However, the involvement of ICAM5 in the neuropathology and spine maturation in FXS remains unclear.

In this study, we aim to examine a possible link between ICAM5 and FMRP in FXS, and to further investigate the molecular detail and the pathological consequences. We confirmed that ICAM5 mRNA is a target of FMRP. In the FXS mouse model, ICAM5 expression is aberrantly increased, correlated with the developmental delay of spine maturation and the concomitant cognitive impairment, and the reduction of ICAM5 expression rescues the behavioral disorders in *Fmr1* KO mice.

## Materials and Methods

### 

#### 

##### Animals.

*Fmr1* knock-out (KO; FVB.129P2-Pde6b^+^Tyr^c-ch^ *Fmr1*^tm1Cgr^/J) and wild-type (WT; FVB.129P2-Pde6b^+^ Tyr^c-ch^/AntJ) mice were purchased from The Jackson Laboratory. All procedures that involved animals were performed in accordance with a protocol approved by the Wuhan University of Science and Technology Animal Research Committee. Male mice were used in all experiments.

##### Western blotting analysis.

The protein level from each selected telencephalic region was measured by Western blot analysis, as previously described (Zeng et al., 2012). Primary antibodies used in this study were goat polyclonal anti-ICAM5 (1:1000; catalog #2507S, Santa Cruz Biotechnology), rabbit polyclonal anti-ICAM5 (1:1000; catalog #ab232785, Abcam), and rabbit polyclonal anti-FMRP (1:1000; catalog #4317S, Cell Signaling Technology).

##### Primary neuron culture, transfection, and morphometrical analysis.

Primary mouse neuronal cultures were obtained from the cerebral cortex of embryos [embryonic day 17 (E17) to E18; Hozumi et al., 2003). For neuron transfection, lentiviral vectors were used with 1 × 10^8^ transduction units/ml (multiplicity of infection, 10). To suppress and overexpress FMR1, we used GV118 (Shanghai Genechem) with the target sequence 5′-ACGAAACTTAGTAGGCAAA-3′ and the GV303 vector (Shanghai Genechem) with full-length FMR1, respectively. After 24 h of transfection, the neurons were cultured for 2 d for protein measurement, and for 8 d for morphological observation of the dendritic spines. Dil staining was performed as described previously (Cheng et al., 2014), and dendritic spines were examined using an FV1000 confocal microscope (FluoView1000, Olympus). Spine head width and length of dendritic protrusions were measured by ImageJ. Only spines within 100 μm cell bodies were evaluated.

##### Quantitative real-time PCR.

We evaluated the mRNA level of FMR1 and ICAM5 in *Fmr1* KO versus WT mice by quantitative real-time PCR (qRT-PCR). Total RNA was extracted by Invitrogen TRIzol Reagent (Thermo Fisher Scientific) and subsequently synthesized into single-strand cDNA using Invitrogen Superscript II reverse transcriptase (Thermo Fisher Scientific). The cDNA amplification was performed using SYBR Premix Ex Tap (Tli RNaseH Plus, Takara) on the Bio-Rad CFX96 system (Chen et al., 2018). Genes and forward/reverse primers used for qRT-PCR are as follows: β-actin: forward, CTCTTTTCCAGCCTTCCTTCTTG; reverse, AGAGGTCTT TACGGATGTCAACG; FMR1: forward, ATCGCTAATGCCACTGTTCTTT; reverse, CGACCCATTCCTTG ACCATC; ICAM5: forward, AGAACAGGAAGGCACCAAACAG; reverse, CTGG CTCACTCAAAGTCAGAAGAG; and U1 snRNA: forward, GGGAGATACCATGATC ACGAAGGT; reverse, CCACAAATTATGCA GTCGAGTTTCCC.

##### Linear sucrose gradient fractionation.

Three-week-old mouse hippocampus lysates and neurons were submitted to Panjin Fengrui Bio-Technology and Yusen Biotechnology for ribosome-bound mRNA testing.

##### Golgi impregnation procedure and spine analysis.

The Golgi staining method was performed with the FD Rapid GolgiStain Kit (FD Neurotechnologies) as previously described in studies by Tian et al. (2015) and Gao et al. (2016). Categories of spine morphology were identified as follows: mushroom-shaped spine (spine with a large bulbous head; the diameter of spine head minus the diameter of the spine neck, ≥1.5 μm); thin spine (filopodia-like protrusion, diameters of spine and neck are nearly equal, and spine length is greater than spine width); and stubby spine (short spine without a well defined spine neck).

##### RNA-binding protein immunoprecipitation and RNA immunoprecipitation sequencing cDNA library construction.

RNA-binding protein immunoprecipitation (RIP) experiments were conducted using the Magna RIP RNA-Binding Protein Immunoprecipitation Kit (Millipore) according to the manufacturer instructions (Moradi et al., 2016). For RIP-sequencing analysis, two types of biological replicates of total RNA samples were obtained, one from the input group (lysate) and one from the FMRP group (lysate incubated with anti-FMRP antibody and magnet). Biological replicates were then submitted to Novogene for sequencing. The cDNA library synthesis from total RNA combined with FMRP was performed according to the manufacturer protocols (Illumina). To identify FMRP binding sites on ICAM5 mRNA, we used Crosslinking-Immunprecipitation and High-Throughput sequencing (HITS-CLIP) with MEME and Dreme software (Bailey et al., 2009) to analyze and then Tomtom software (Gupta et al., 2007) to compare the existing motifs in the database.

##### Stereotactic surgery and virus injection.

Mice were anesthetized with isoflurane and placed in a stereotaxic apparatus (item #68030, RWD). To suppress ICAM5 expression in dentate gyrus (DG), AAV-ICAM5 shRNA-EGFP (BrainVTA) was injected into either DG (coordinates: the anterior–posterior, −1.70 mm; lateral, ±1.20 mm) of 1-month-old *Fmr1* KO mice. Four weeks after virus injection, mice were submitted to the following tests.

##### Slice physiology.

Slices (400 μm) were obtained from virus- or vector-infected *Fmr1* KO male mice. Mice were anesthetized with isoflurane and rapidly decapitated, and brains were quickly removed and transferred into ice-cold artificial CSF (ACSF) containing the following (in mm): 124 NaCl, 26 NaHCO_3_, 3 KCl, 2 CaCl_2_, 1 MgCl_2_, 1.25 KH_2_PO_4_, and 10 glucose, pH 7.4 (oxygenated with 95% O_2_ and 5% CO_2_). Slices were cut in ice-cold dissection solution with a vibrating blade microtome and incubated in an interface chamber for 0.5 h at 34–36°C and 1 h at room temperature. The 64-channel multielectrode (MED64) system (Alpha MED Sciences) was used to record field EPSPs (fEPSPs), as previously described (Chin-Wei et al., 2006; Chang et al., 2015). Slices were placed on the top of the 8 × 8 microelectrode arrays (MED-P515A probe), incubated with continuously infused ACSF at 34°C at the flow rate of 2 ml/min. Stimuli were delivered to the slice via one selected site, and the intensity was calculated by 50% of the maximal synaptic response determined by input–output curves, while the response microelectrodes were used to record the fEPSPs. A LTD was induced with low-frequency stimulation (1 Hz, 900 pulses).

Spontaneous EPSCs (sEPSCs) were collected from granular cells in the hippocampal DG using conventional whole-cell recording techniques with a Multiclamp 700B amplifier connected to a 1550B analog-to-digital board and Clampex 10 program suite (Molecular Devices). Intracellular solution contained the following (in mm): 150 KCl, 10 HEPES, 4Mg2ATP, 0.5 NaGTP, 10 phosphocreatine, and 0.2% biocytin or 135 K-gluconate, 15 KCl, 5 NaCl, 0.5 EGTA, 10 HEPES, 2Mg2ATP, and 0.2% biocytin, pH 7.3 and 270–290 mOsm and 3–7 MΩ resistance. sEPSC were collected in voltage-clamp mode at a holding potential value of −70 mV at a temperature of 30–32°C. Using the template-based analysis feature of Clampfit 10.0, sEPSC events were collected and analyzed. Access resistance was monitored throughout the experiment, and data from experiments were excluded if the access resistance or the input resistance changed >20% during the experiment.

##### Behavioral tests.

Morris water maze (MWM) test and fear-conditioning paradigm test were used to assess spatial learning, fear learning, and memory, as previously described (Zeng et al., 2012). Open field (OF) test and elevated plus maze (EPM) tests were used to characterize the locomotor and anxiety-like behaviors of the mice (Gao et al., 2016).

The social interaction test was performed with a three-chamber device, as previously reported (Tabuchi et al., 2007). After 5 min of habituation, the testing mouse was first placed in the middle chamber, a strange mouse S1 that had no prior contact with the testing mouse was placed in right-side chamber, while the left-side chamber was empty in session 1. Then the testing mouse was tested in session 2 with another strange mouse (S2), but in the left chamber, while the right chamber was empty. In session 3, the testing mouse had options between the first already-investigated S1 and a new strange mouse (S3). Both doors to the side chambers were then unblocked, and the subject mouse was allowed to explore the entire social test box for 10 min in each session.

##### Experimental design and statistical analysis.

*Fmr1* KO and age-matched WT male mice were used in all experiments. In all cases, four or more animals of the same age and genotype were used for each parameter. Each neuron was considered as an individual data point for dendritic morphological and electrophysiological experiments. For Western blot and behavioral test, *n* values (number of animals) were reported. Statistical analyses were performed using Microsoft Excel and SPSS 16.0 software. For the comparison between two groups, data were analyzed with an unpaired two-tailed Student's *t* test. One-way ANOVA and two-way ANOVA followed by Bonferroni's *post hoc* tests were performed for multiple comparisons. The statistical test and sample size (*n*) for each experiment were specified in the figure legends. Data were presented as the mean ± SEM, with *p* < 0.05 being considered statistically significant.

## Results

### Lack of FMRP in *Fmr1* KO mice results in changed ICAM5 expression and immature thin spines

**Figure 1.**
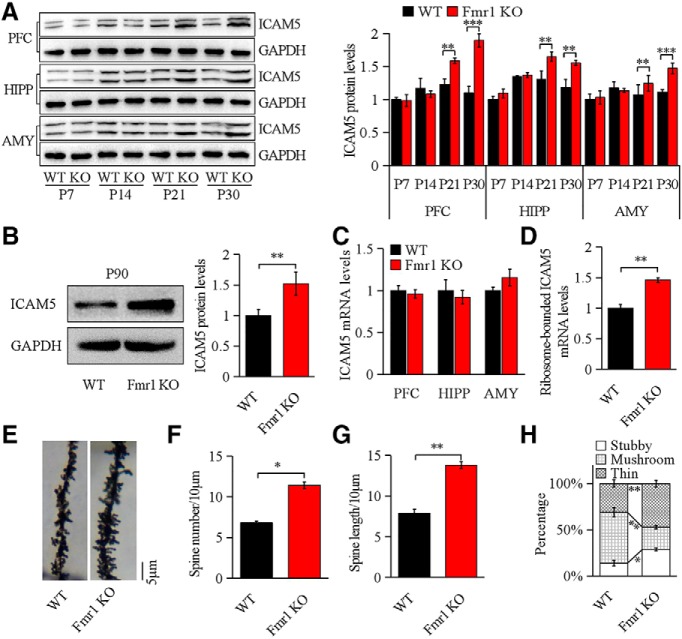
Increased ICAM5 expression and immature spines in *Fmr1* KO mice. ***A***, Representative Western blotting images and quantification of ICAM5 expression from three brain regions [the PFC, hippocampus (HIPP), and amygdala (AMY)] during postnatal developmental stage. ***B***, Representative Western blotting images and quantification of ICAM5 expression at P90 in the hippocampus. ***C***, Quantification of ICAM5 mRNA with qRT-PCR in prefrontal cortex, hippocampus, and amygdala in *Fmr1* KO and WT at P21. ***D***, Relatively more ribosome-bounded ICAM5 mRNA was found in 21-d-old *Fmr1* KO mice. ***E***, Representative photograph of Golgi stained apical dendrites of *Fmr1* KO and WT neurons. ***F***, ***G***, Increased spine number and prolonged spine length in *Fmr1* KO neurons after Golgi staining (per 10 μm dendrite). ***H***, Dendritic spine classification by morphology in *Fmr1* KO and WT hippocampus. *N* = 480 terminals for WT; and *n* = 493 terminals for *Fmr1* KO (6 mice/group). Data are presented as the mean ± SEM. ***p < 0.001, **p < 0.01, *p < 0.05 by unpaired two-tailed Student's *t* test.

We first examined the expression of ICAM5 expression in the developing prefrontal cortex, hippocampus, and amygdala (Fig. 1*A*) in WT and *Fmr1* KO mice. In *Fmr1* KO mice, ICAM5 expression was increased during late brain development from postnatal day 21 (P21) to P90 when the mice developed a mature hippocampus (Fig. 1*A*,*B*), while in prefrontal cortex and amygdala, the expression of ICAM5 was also found to be increased since P21 (Fig. 1*A*). At P21, ICAM5 expression in *Fmr1* KO mice was increased up to 20 ± 1.9% (*p* = 0.0034) relative to that in WT hippocampus, up to 27 ± 1.5% in the prefrontal cortex, and 25 ± 1.9% in the amygdala. However, the ICAM5 mRNA level at P21 was unchanged in the prefrontal cortex, the hippocampus, or the amygdala (Fig. 1*C*), suggesting a translational disorder of ICAM5 mRNA in *Fmr1* KO mice. In accordance with this, polyribosome fractionation analysis showed that ribosome-bounded ICAM5 mRNA was significantly increased (39 ± 1.8%, *p* = 0.0051) in 3 weeks *Fmr1* KO hippocampus (Fig. 1*D*) compared with WT, further suggesting a potential role of FMRP on ICAM5 translation.

As ICAM5 negatively regulates dendritic spine maturation and facilitates immature spine formation (Conant et al., 2011), we examined the dendritic spines of Golgi-impregnated *Fmr1* KO and WT neurons. As shown in Figure 1, *F* and *G*, spine number and length measurements were significant higher in KO neurons compared with those in WT hippocampus neurons at P21 (40 ± 2.8%, *p* = 0.039; 43 ± 1.7%, *p* = 0.0032). The same tendency was also found in the prefrontal cortex and the amygdala (data not shown), indicating a spine overproduction in FXS. Concomitantly, during P21 and the later brain development, WT spines exhibited a steady state, whereas *Fmr1* KO spines remained at prepruning levels. As shown in Figure 1*H*, at P21, the percentages of immature thin and stubby spines in the *Fmr1* KO hippocampus were significantly higher than that in WT hippocampus (26 ± 3.1%, *p* = 0.0071), while mature mushroom-shaped spines were significantly decreased (34 ± 2.7%, *p* = 0.0051). These results showed a developmental delay in the downregulation of spine turnover and in the transition from immature to mature spine subtypes, which matches the time course and reported role of ICAM5 overexpression in the literature (Raemaekers et al., 2012).

### Reduction of ICAM5 normalizes dendritic spine abnormalities in *Fmr1 KO* neurons

**Figure 2.**
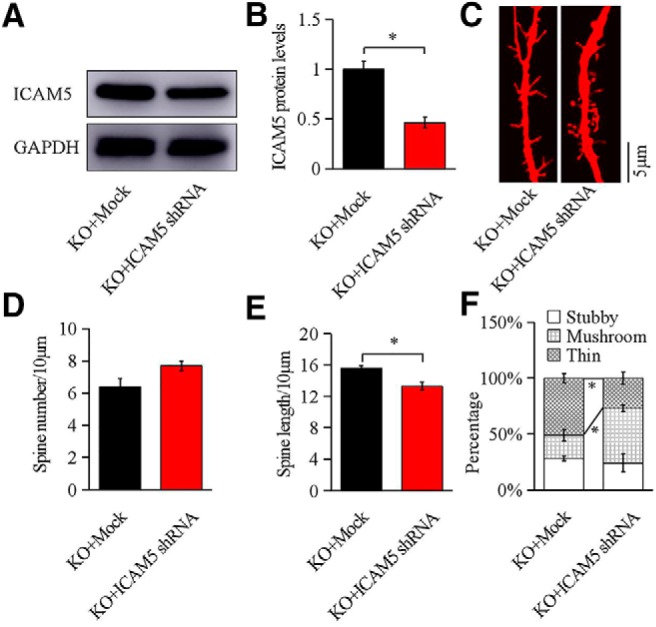
ICAM5 shRNA affected dendritic morphology in cultured *Fmr1* KO neurons. ***A***, ***B***, Representative Western blotting images of ICAM5 and quantification of ICAM5 expression after transfection. ***C***, Representative dendritic segments from *Fmr1* KO neurons after transfection (Dil staining). ***D***, ***E***, Statistical analyses of the number and length of spines per 10 μm dendrites. ***F***, Morphology analyses for thin, mushroom, and stubby-shaped spines. *N* = 506 terminals for KO + Mock and *n* = 500 terminals for KO + ICAM5 shRNA (8 mice/group). Data are presented as the mean ± SEM. *p < 0.05 by unpaired two-tailed Student's *t* test.

To verify the involvement of ICAM5 in dendritic spines, we suppressed ICAM5 expression in *Fmr1* KO neurons and compared with neurons transfected empty lentiviral vectors (Mock). As shown in Figure 2, *A* and *B*, ICAM5 protein level in *Fmr1* KO neurons was significantly decreased by ICAM5 shRNA (45 ± 1.6%, *p* = 0.0114), validating ICAM5 protein suppression. The total spine number was not modified by ICAM5 suppression (Fig. 2*C*,*D*). However, the thin spines in ICAM5 shRNA *Fmr1* KO neurons were fewer than those in controls (39 ± 2.4%, *p* = 0.0143; Fig. 2*C*,*F*), and no significant changes in stubby spines, while the mushroom spines increased (23 ± 2.6%, *p* = 0.0289), which suggests that ICAM5 suppression attenuated the aberrant maturation of dendritic spines in *Fmr1* KO neurons. In addition, the mean length of all spine types was significantly decreased (18 ± 1.8%, *p* = 0.0153; Fig. 2*E*). Together, these data demonstrate that the overexpression of ICAM5 in FXS is positively related to the abnormal dendritic spine length and maturation in *Fmr1* KO neurons.

### FMRP affects ICAM5 protein expression and dendritic morphology in cultured neurons

**Figure 3.**
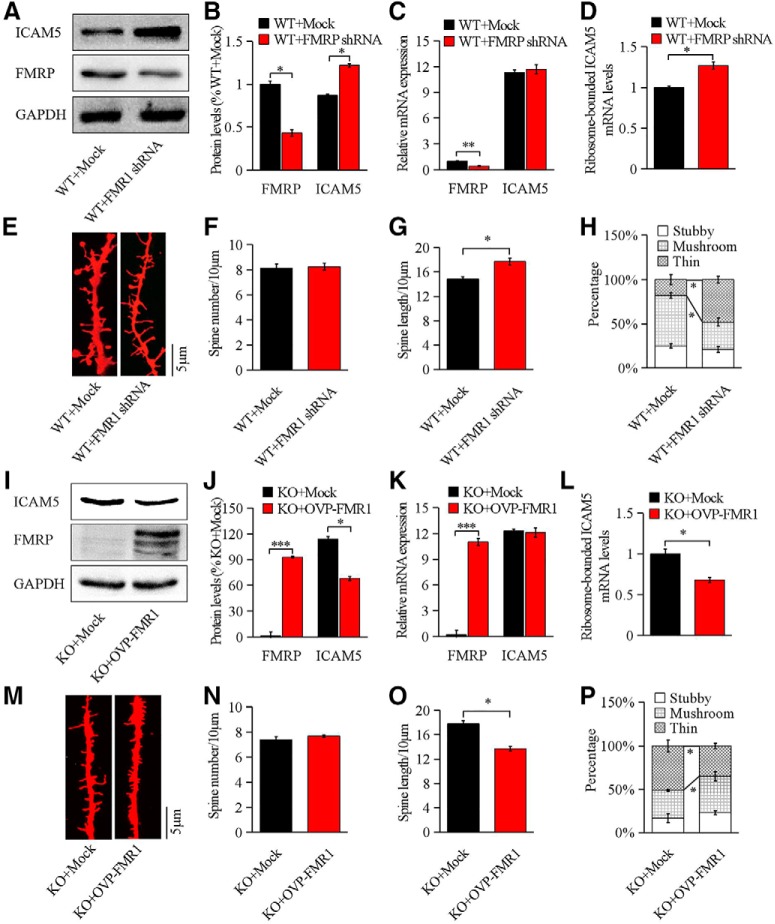
FMR1 affected ICAM5 protein expression and dendritic morphology in cultured WT and KO neurons. ***A–H***, FMR1 shRNA transfected cultured WT neurons. ***A***, ***B***, Raised ICAM5 and reduced FMRP expression after FMR1 shRNA transfection. ***C***, ***D***, qRT-PCR analyses of total and ribosome-bounded ICAM5 mRNA, respectively. ***E–G***, Representative dendritic segments from cultured neurons (***E***; Dil staining) and statistical analyses of spine number/length (***F***, ***G***) per 10 μm dendrites. ***H***, Dendritic spine classification by morphology after FMR1 interference. *N* = 496 terminals for WT + Mock, *n* = 470 terminals for WT+ FMR1 shRNA (8 mice/group). ***I–P***, FMR1 overexpression transfected cultured KO neurons. ***I***, ***J***, Representative Western blotting images and quantification of ICAM5 and FMRP. ***K***, ***L***, qRT-PCR analyses of total and ribosome-binding ICAM5 mRNA, respectively. ***M–O***, Representative dendritic segments from cultured neurons (Dil staining) and statistical analyses of spine number/length per 10 μm dendrite. ***P***, Dendritic spine classification by morphology after FMR1 overexpression. *N* = 490 terminals for KO + Mock, and *n* = 524 terminals for KO + OVP-FMR1 (8 mice/group). Data are presented as the mean ± SEM. ***p < 0.001, **p < 0.01, *p < 0.05 by unpaired two-tailed Student's *t* test.

To identify whether the increased ICAM5 protein level in *Fmr1* KO mice resulted from the loss of FMRP, we modified the FMRP protein levels in WT and KO neurons and examined the alterations in ICAM5 expression and dendritic morphology. Two days after transfection with lentiviral vector, FMRP and ICAM5 expression was tested by Western blotting. As shown in Figure 3, the ICAM5 protein level was negatively correlated with the FMRP level. In WT neurons, ICAM5 was significantly increased (30 ± 1.5%, *p* = 0.0342; Fig. 3*A*,*B*) by FMR1 interference, and ICAM5 mRNA was kept unchanged (Fig. 3*C*; *p* = 0.0924); whereas FMR1 overexpression significantly decreased ICAM5 in KO neurons (35% ± 1.2%, *p* = 0.0272; Fig. 3*I*,*J*), without affecting ICAM5 mRNA (Fig. 3*K*; *p* = 0.0853). However, polyribosome fractionation analysis showed that ribosome-bounded ICAM5 mRNA was significantly increased (23 ± 1.3%, *p* = 0.0283) after FMR1 interference in WT neurons (Fig. 3*D*), and the consistently FMR1 overexpression resulted in reduced ribosome-bounded ICAM5 mRNA in KO neurons (32 ± 2.0%, *p* = 0.0121; Fig. 3*L*), indicating the role of FMRP on ICAM5 translation.

To further examine the translational effect of FMR1 on dendritic morphology, we observed spine morphology 7 d after FMR1 shRNA or Ovp-FMR1 lentiviral transfection. Spine length was significantly elongated in FMR1 knock-down WT neurons (28 ± 2.9%, *p* = 0.0204; Fig. 3*E*,*G*) and shortened in FMR1 overexpression KO neurons (18 ± 3.1%, *p* = 0.0294; Fig. 3*M*,*O*). Furthermore, the percentage of thin spines in FMR1 knock-down WT neurons increased by 47 ± 2.7% (*p* = 0.0113) over that in controls (Fig. 3*H*), while the percentage of mushroom spines was significantly reduced (44 ± 3.3%, *p* = 0.0192). By contrast, the percentage of thin spines decreased in FMR1-overexpressed KO neurons (33% ± 2.8%, *p* = 0.0221, Fig. 3*M*,*P*) and the mushroom spines increased (29 ± 3.3%, *p* = 0.0382). These results indicate that FMRP-related ICAM5 protein expression corresponds with dendritic spine morphology, although the total number of dendritic spines was not changed (Fig. 3*F*,*N*). Given the role of ICAM5 in dendritic spine formation and maturation, as reported in the literature (Raemaekers et al., 2012) and confirmed in this study (Fig. 2), we hypothesize that FMRP directly affects ICAM5 expression, which consequently influences spine morphology.

### FMRP directly binds to ICAM5 mRNA *in vitro*

**Figure 4.**
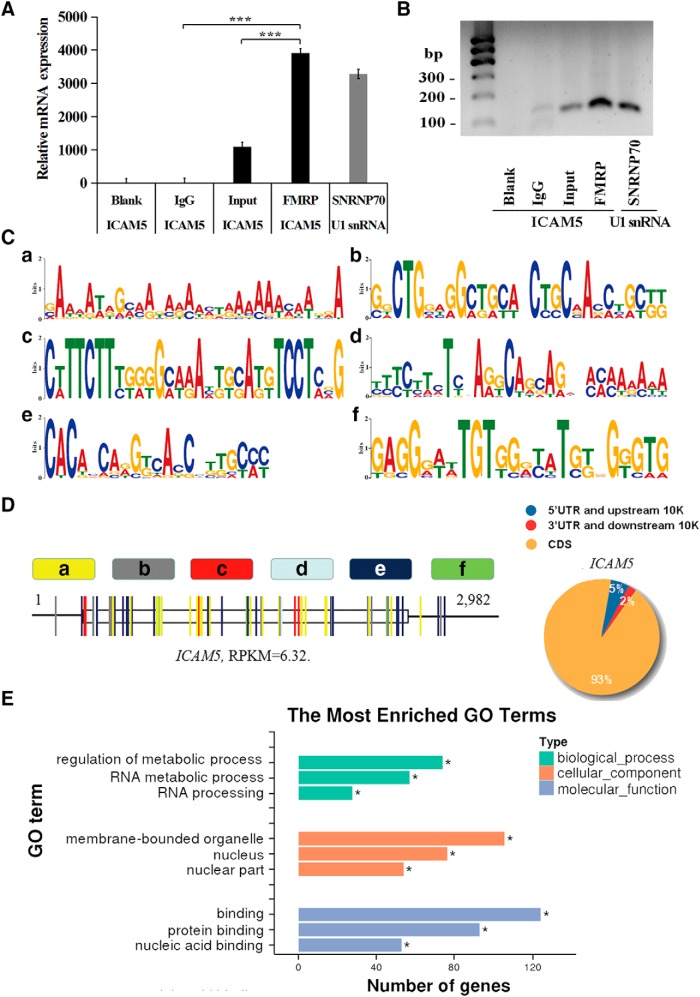
FMRP binding sites on ICAM5 mRNA determined by sequence analysis. ***A***, ICAM5 mRNA was found in the FMRP-extracted group by qPCR. Negative control: Blank and IgG groups. Positive control: ICAM5 mRNA in lysate input group and U1 snRNA in SNRNP70 group. ***p < 0.001 by one-way ANOVA with a Bonferroni's *post hoc* test. ***B***, DNA gel electrophoresis of the qPCR products of ***A***. ***C***, Six major ICAM5 RNA recommended segments (***a–f***) that were inferred to be the FMRP binding sites. ***D***, Left, Distribution of the six FMRP binding motifs (***a–f***) across the representative ICAM5 mRNA. Open boxes and bold lines indicate CDS and untranslated regions (UTRs), respectively. Right, Occurrence frequency of the six motifs in CDS and UTRs. ***E***, The top three GO terms enriched in FMRP target transcripts and their GO categories; *n* = 3. *p_adj_ < 0.05 (Benjamini-Hochberg).

To determine whether ICAM5 mRNA is an FMRP target, we tested the FMRP–ICAM5 mRNA interaction *in vitro* with RIP followed by qRT-PCR and DNA gel electrophoresis. As shown in Figure 4*A* and *B*, ICAM5 mRNA appeared in the FMRP antibody-extracted group and the total input group, but not in the IgG extracted or Blank (no template PCR control) groups, indicating a direct binding of FMRP to ICAM5.

HITS-CLIP results show that 10 FMRP-connected mRNA motifs were frequently detected, and 6 of them were highly matched with the ICAM5 mRNA sequence (Fig. 4*C*,*D*). Namely, they were AGACMMM, RAAAAWC, ARAAAAW, CACAGCA, SCVAVCH, and TSKGGKC (M = A/C, *R* = A/G, W = A/T, K = G/U, S = G/C, V = G/A/C, and H = A/T/C). Within the ICAM5 mRNA, 93% of the six motifs appeared within the coding sequence (CDS; Fig. 4*D*). Most of them are located very close to one another, and some are overlapped (data not shown). Gene Ontology (GO; http://www.geneontology.org/) was used to gain insight into the biological functions encoded by the FMRP target transcripts. As seen in Figure 4*E*, the FMRP target transcripts were mainly located around the nucleus- and membrane-bounded organelles, suggesting the direct biological modulating role of FMRP on ICAM5.

### Genetic reduction of ICAM5 in DG corrects behavioral deficits in FXS

To evaluate the functional relevance of elevated ICAM5 expression in FXS, we examined spatial and fear memory and exploratory and anxiety-like behaviors in *Fmr1* KO and ICAM5 knock-down (AAV-ICAM5 shRNA-EGFP) *Fmr1* KO mice, and compared their performance with WT, ICAM5 knock-down WT, and adenovirus empty vector (AAV-EGFP)-transfected *Fmr1* KO and WT mice.

**Figure 5.**
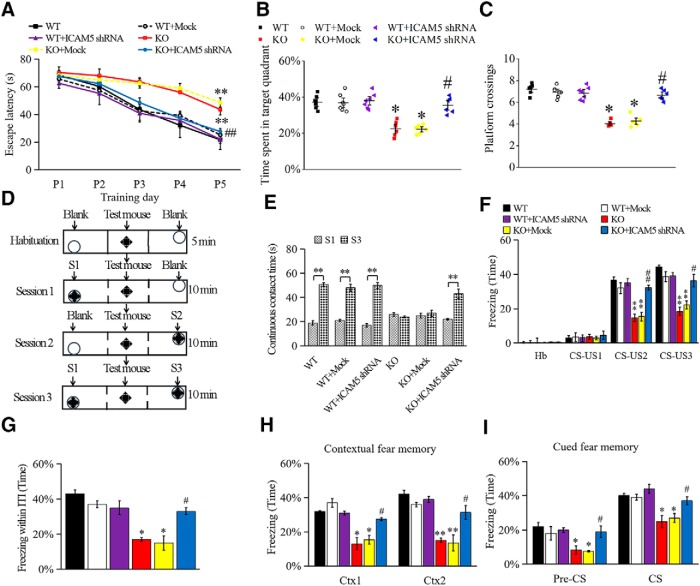
ICAM5 knockdown rescued the impaired behavioral performances in *Fmr1* KO mice. ***A–C***, MWM test. ***A***, Mean escape latency of reaching the submerged platform during the training period. ***B***, ***C***, Time spent in target quadrant and platform crossings after training. ***D***, ***E***, Social interaction test. ***D***, The scheme of the three-chamber social interaction test. ***E***, Contact time of the testing mouse with a strange mouse. ***F–I***, Fear-conditioning paradigm test. ***F***, Percentage of freezing during the period of associative conditioned stimulus–unconditioned stimulus (CS-US) pairing. ***G***, Averaged freezing time during the intertrial intervals (ITIs) of the testing session. ***H***, ***I***, Contextual fear conditioning and cued fear conditioning 24 h after CS-US pairing stimulation. *N* = 6 for WT, *n* = 7 for WT + Mock, *n* = 7 for WT + ICAM5 shRNA, *n* = 6 for KO, *n* = 6 for KO + Mock, and *n* = 6 for KO + ICAM5 shRNA. Data are presented as the mean ± SEM. Two-way ANOVA was used with a Bonferroni's *post hoc* test for statistical analysis. **p < 0.01, *p < 0.05 compared with WT; ##*p* < 0.01, #*p* < 0.05 compared with *Fmr1* KO mice.

We first evaluated spatial memory with the hidden platform MWM. During the training sessions, KO mice showed significantly longer escape latencies than WT mice, but the latency was shortened in ICAM5 shRNA KO mice (Fig. 5*A*). After 5 d of training, spatial memory retention was evaluated by removing the hidden platform. KO mice (39 ± 2.3%, *p* = 0.0255, vs WT mice) showed no preference for the correct quadrant, whereas both the WT and ICAM5 shRNA KO groups spent more time in the correct quadrant (Fig. 5*B*) and crossed the previous hidden platform location more frequently (Fig. 5*C*), which suggested the impairment of spatial memory performance of *Fmr1* KO mice and correction by the reduction of ICAM5 expression. WT mock and ICAM5 shRNA WT mice exhibited no difference from WT. In addition, at the visible-platform test, all groups of mice showed comparable escape latency and swimming speed (data not shown), suggesting a comparable motor and visual function among the different mice, and no change in vision or swimming speed was found that could influence the behavioral tests.

The social interaction test was performed as shown in the schematic drawing (Fig. 5*D*). After habituation, all mice presented a preference for strange mouse, S1 in session 1 and for S2 in session 2, over Blank (data not shown). However, in session 3, WT and ICAM5 shRNA KO mice showed a preference for new strange mouse S3, instead of re-put mouse S1 (Fig. 5*E*; 54 ± 2.9%, *p* = 0.0130; 50 ± 2.0%, *p* = 0.0183). However, KO and KO Mock groups did not show the preference (*p* = 0.6364 and *p* = 0.4272), indicating reduced social interaction and memory in *Fmr1* KO mice and reversed the effect of ICAM5 reduction. WT mock and ICAM5 shRNA WT mice showed no difference from WT.

For the fear-conditioning learning test, during the second and third tone–shock pairs (Fig. 5*F*), freezing time was decreased ∼50 ± 3.5% (*p* = 0.0065) in KO mice relative to WT mice. During the intermission, KO mice also showed less freezing time, suggesting impaired fear memory (Fig. 5*G*; *p* = 0.0124). A day after the contextual test, KO mice continued to exhibit less freezing time (Fig. 5*H*; *p* = 0.0219) when delivered into the contextual fear-conditioning environment. There was also decreased memory consolidation for cued fear conditioning (Fig. 5*I*; *p* = 0.0153) 2 h after the contextual fear-conditioning test. All above impaired memory-related behaviors were improved in ICAM5 shRNA KO mice compared with KO mice, during the contextual test (Fig. 5*G*; *p* = 0.0166), in the conditioning environment (Fig. 5*H*; *p* = 0.0138), and cued fear condition (Fig. 5*I*; *p* = 0.0247), whereas WT mock and ICAM5 shRNA WT mice had no difference compared with WT mice.

**Figure 6.**
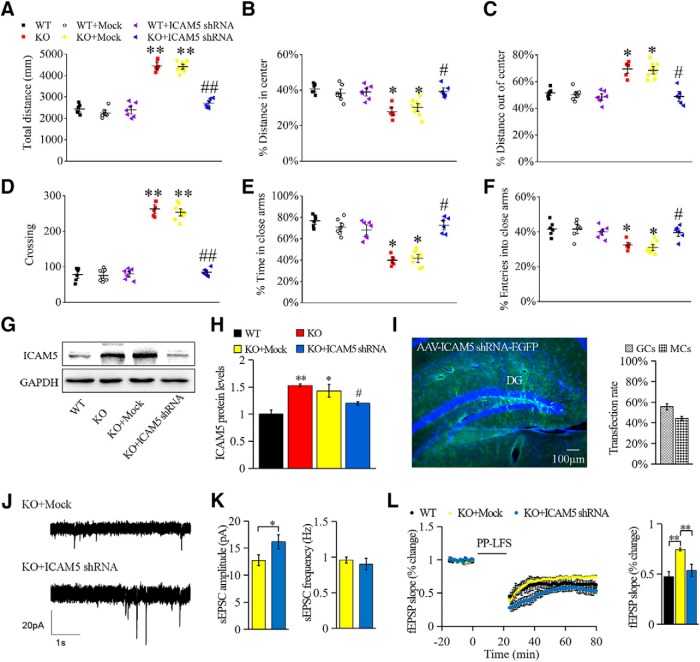
ICAM5 knockdown improved the locomotor or anxiety-like behaviors in *Fmr1* KO mice. ***A–D***, Open-field test. ***A***, Overall distance traveled during the 5 min open-field test. ***B***, ***C***, Percentages of the distance in the center area over total distance (***B***) and peripheral distance over total distance (***C***). ***D***, Number of times across the cells per minute. ***E***, ***F***, Elevated plus maze. ***E***, Times entered into closed arms over total entries into both closed and open arms. ***F***, Percentage of time spent in closed arms. *N* = 6 for WT, *n* = 7 for WT + Mock, *n* = 7 for WT + ICAM5 shRNA, *n* = 6 for KO, *n* = 9 for KO + Mock, and *n* = 6 for KO + ICAM5 shRNA. **p < 0.01, *p < 0.05 compared with WT; ##*p* < 0.01, #*p* < 0.05 compared with *Fmr1* KO mice. ***G***, ***H***, ICAM5 expression in hippocampus after ICAM5 knockdown. ***I***, Confocal images (left) of the KO dentate gyrus transfected with ICAM5 shRNA virus (green) and DAPI staining nucleus (blue), while the percentages of transfected GCs and MCs are shown on the right. *N* = 7. ***J***, ***K***, Example and statistical analyses of sEPSC amplitude and frequency in EGFP expressed neurons in KO mock and KO ICAM5 shRNA mice. *N* = 18 neurons for KO + Mock and *n* = 14 neurons for KO + ICAM5 shRNA (4 mice/group). ***L***, Representative traces before and after LTD induction (left) and mean fEPSP slopes averaged 50–60 min after LTD induction (right). *N* = 7 for WT, *n* = 6 for KO + Mock, and *n* = 5 for KO + ICAM5 shRNA. Data are presented as the mean ± SEM; unpaired two-tailed Student's *t* test and two-way ANOVA with a Bonferroni's *post hoc* test were used for statistical analysis. **p < 0.01, *p < 0.05.

In addition, the mice were then tested for exploratory behavior in the OF test (Fig. 6*A–D*). *Fmr1* KO mice exhibited longer total travel distance (Fig. 6*A*; 46 ± 2.3%, *p* = 0.0053); however, a lower percentage of total distance inside the center than with WT mice (Fig. 6*B*; 39 ± 2.8%, *p* = 0.0211), and a greater percentage of the total distance out of center (Fig. 6*C*; 21%, *p* = 0.0283), and a greater number of times across the edges (Fig. 6*D*; 58 ± 1.9%, *p* = 0.0315). The results suggested a significantly elevated exploration activity and unconditioned anxiety-related behavior in *Fmr1* KO mice, which was reversed by DG ICAM5 knockdown for total travel distance (Fig. 6*A*; 38 ± 3.6%, *p* = 0.0032), center distance (Fig. 6*B*; 24 ± 2.1%, *p* = 0.0455), out-of-center distance (Fig. 6*C*; 21%, *p* = 0.0365), and times across the edges (Fig. 6*D*; 57 ± 4.4%, *p* = 0.0024). In the EPM test, *Fmr1* KO mice also showed impaired anxiety-like behavior. *Fmr1* KO mice displayed less time spent in and fewer times entering the closed arms (Fig. 6*E*,*F*; 35 ± 3.7%, *p* = 0.0145; and 18 ± 2.4%, *p* = 0.0426), which was also recovered in the ICAM5 shRNA group (28 ± 1.6%, *p* = 0.0211 and 16 ± 1.5%, *p* = 0.0421). Instead, WT mock and ICAM5 shRNA WT mice showed no difference from WT mice in the OF and EPM test.

After the behavioral tests, mice were killed, and their brains were collected for detecting transfection efficiency and ICAM5 expression. The ICAM5 protein level in the *Fmr1* KO hippocampus was significantly decreased by ICAM5 shRNA intervention (43 ± 1.1%; Fig. 6*G*,*H*). Moreover, the large amount of EGFP expression showed that AAV-ICAM5 shRNA-EGFP was successfully transfected into the dentate gyrus (Fig. 6*I*), validating ICAM5 protein suppression. Surprisingly, both granule cells (GCs) and mossy cells (MCs) were transfected with a relative rate of 1.32 ± 0.15, accounting for 56.94 ± 2.96% and 43.06 ± 2.32% of total transfected cells.

To investigate the electrophysiological modification after ICAM5 suppression, sEPSC was recorded on the EGFP-expressed neurons after Mock or ICAM5 shRNA injection. Compared with the Mock group, ICAM5 shRNA intervention significantly increased the amplitude of sEPSCs (Fig. 6*J*,*K*; *p* = 0.0160) without changing the frequency (Fig. 6*J*,*K*) in GCs, which was consistent with the results of morphological maturation of the dendritic spines (Fig. 2*F*). Besides, ICAM5 shRNA intervention significantly rescued the impaired synaptic plasticity, where the classic abnormal LTD is improved (Fig. 6*L*; *p* = 0.0032). Although, we did not observe significant difference in LTP between *Fmr1* KO and WT mice (data not shown), which is also found by many groups based on different experimental conditions (Li et al., 2002; Larson et al., 2005).

## Discussion

The present study verified for the first time the novel FMRP target ICAM5 mRNA and explored its contribution to spine abnormalities and behavioral defects in FXS. We found that the loss of FMRP relieves its direct binding with ICAM5 mRNA and induces ICAM5 overexpression, which is translationally related to dendritic spine morphological abnormalities in *Fmr1* KO neurons. Viral intervention of ICAM5 expression in DG reverses the cognitive deficits in the FXS mouse model *Fmr1* KO mice, demonstrating the therapeutic value of ICAM5 for treating cognitive dysfunctions in FXS. To our knowledge, this is the first study that detected the role of ICAM5 in cognitive function *in viv*o.

A salient neuropathological defect in FXS is dendritic spine dysgenesis, but its underlying mechanism is still unclear. Previous reports indicate that FMRP regulates the expression of synaptic proteins including PSD-95, CaMKIIα, and MAP1B (Lu et al., 2004; Hou et al., 2006; Zalfa et al., 2007; Kao et al., 2010; Darnell, 2010; McMahon and Rosbash, 2016). ICAM5 is also reported to promote spine outgrowth via homophilic binding (Tian et al., 2000; Recacha et al., 2014), via ICAM5–ERM (ezrin/radixin/moesin) interaction and ectodomain interaction with β1 integrins (Yang, 2012). The homophilic adhesion of ICAM5 mediates the induction of dendritic outgrowth (Tian et al., 2000), since the ICAM5 cytoplasmic region binds ERM family proteins that link membrane proteins to actin cytoskeleton (Furutani et al., 2007), while the ICAM5 and β1 interaction via the two first Ig domains stimulates cofilin phosphorylation and facilitates matrix metalloproteinase-dependent spine maturation (Conant et al., 2011; Ning et al., 2013). However, ICAM5, the direct negative regulator of dendritic spine maturation has never been examined in FXS. ICAM5 is expressed at low levels in embryos but rapidly increased after birth when large numbers of synapses are formed (Matsuno et al., 2006). During spine maturation, ICAM5 expression gradually decreases (Matsuno et al., 2006), which is also observed in our results with age. However, in *Fmr1* KO mice, since postnatal day 21, ICAM5 was more abundantly expressed than in WT mice, which is consistent with the timing of increased thin spines and decreased mushroom spines. Besides, reduced ICAM5 expression resulted in spine maturation (Fig. 2*F*) and synaptic response (Fig. 6*J–L*) in *Fmr1* KO neurons, indicating the involvement of ICAM5 in KO neuron spine formation. Regarding the reported effect of ICAM5 in spine pruning and formation (Matsuno et al., 2006), these results indicated a critical role of ICAM5 in FXS spine maturation and brain development. Considering that ICAM5 increases after P21, the alterations in spine length and numbers at P14 indicate the existence of multiple mechanisms in FXS spine abnormality.

FMRP is an RNA-binding protein controlling mRNA translation by promoting its dynamic transport and stalling its translation (Darnell et al., 2011; Darnell and Klann, 2013). Most of the previous studies supported that FMRP binds to a large number of mRNAs (Darnell et al., 2011). Our results showed that ICAM5 expression is excessively expressed in *Fmr1* KO mice, and the expression of FMRP was negatively correlated with the expression of ICAM5. Further experiment indicated that FMRP interacts directly with ICAM5 mRNA, which is consistent with the findings of Darnell et al. (2011). In addition, FMRP bound ICAM5 mRNA predominantly in the coding regions and mainly located around the nucleus and membrane-bound organelles. FMRP is well known for binding proteins and RNA, in turn regulating RNA processing and metabolism (Zhang et al., 2015), which corresponds with the biological modulation of FMRP on ICAM5 mRNA in our study. These results indicate the overexpressed ICAM5 attributed to the loss of FMRP in FXS.

Since both ICAM5 (Mizuno et al., 1997) and FMRP (Hinds et al., 1993) are highly expressed in the cortex and amygdala in WT mice, in addition to hippocampus we also detected ICAM5 expression in the cortex and amygdala in FXS. The results are consistent in all three regions that ICAM5 was excessively expressed in *Fmr1* KO mice, indicating that loss of FMRP-induced ICAM5 overexpression could lead to a potential broad pathological consequence in the mammalian brain and FXS. Indeed, we found remarkable numbers of immature spines in neurons from these three regions. The overexpressed ICAM5 corresponded to the timing of increased thin spines and decreased mushroom spines. All of these results indicate a potential broad role of ICAM5 in the mammalian brain and FXS.

The role of ICAM5 has been well studied for the last decade; however, the therapeutic role of ICAM5 is still unclear (e.g., whether abnormal ICAM5 expression could influence any behavioral disorders *in vivo* is never studied). Our results indicated that ICAM5 knockdown reversed behavioral disorders in *Fmr1* KO mice. Intellectual disability is a characteristic phenotypic feature of FXS, which is not fully understood and cannot be improved by current medication. As reported by many research groups, *Fmr1* KO mice exhibited impaired memory and exploratory and anxiety-like behaviors (Spencer et al., 2005; Baker et al., 2010), which were ameliorated to some degree by lowering ICAM5 expression in DG, a pivotal area connecting amygdala and prefrontal cortex (Zancada-Menendez et al., 2017). Interestingly, ICAM5 suppression did not change the behavior in WT mice. It has been reported that ICAM5-deficient mice showed a decreased density of filopodia and an acceleration of spine maturation *in vitro* and *in vivo* (Matsuno et al., 2006). However, it is unclear whether ICAM5-deficient mice exhibit behavior change, and it could be interesting for future study to evaluate the effect of ICAM5 suppression in normal WT mice. Thus, the overexpression of ICAM5 in postnatal development in *Fmr1* KO mice may be a neurobiological mechanism for FXS pathological phenotypes and a therapeutic target for the treatment of FXS cognitive impairment.

GCs and MCs are two excitatory cell types of the DG. The GC bodies form the granule cell layer, while the MCs are located only in hilus and are the most common cells in polymorphic layer (Amaral et al., 2007). MCs excite or inhibit GCs through direct inputs (Soriano and Frotscher, 1994) or interneuron activation (Scharfman, 1995), and precede GCs in detecting changes and help to expand the range of GC pattern separation (Jung et al., 2019). The understanding of MCs in DG function is limited, but their contributions to behavior have been proposed, including to memory, novelty, and anxiety (Scharfman, 2016). It is still unclear whether MCs are involved in the spine dysgenesis and pathophysiology in FXS. Our results indicated that both GCs and MCs were transfected, with a relative rate of 1.32 ± 0.15. After ICAM5 intervention, the sEPSC amplitude was increased in *Fmr1* KO GCs, probably induced by direct dendritic spine maturation or by the enhanced excited inputs from MCs. Furthermore, ICAM5 intervention in MCs could also contribute to the reversed behavior disorder in KO mice. However, the proportional contribution for GCs and MCs is still unknown. Future experiments using cell type-specific inactivation might directly test MC contribution to FXS.

In summary, our results suggest that ICAM5 is an mRNA target of FMRP and plays a critical role in the spine dysgenesis and pathophysiology of FXS. FMRP could regulate translational events involved in the synthesis of ICAM5 probably via direct binding and concomitantly influences dendritic spine development and disease severity. Genetic ICAM5 intervention attenuated behavioral deficits in *Fmr1* KO mice, which may provide therapeutic benefits in the treatment of FXS cognitive impairment and other NDDs.
